# Secure Aggregation Protocol Based on DC-Nets and Secret Sharing for Decentralized Federated Learning

**DOI:** 10.3390/s24041299

**Published:** 2024-02-17

**Authors:** Diogo Pereira, Paulo Ricardo Reis, Fábio Borges

**Affiliations:** National Laboratory for Scientific Computing, Petrópolis 25651-075, RJ, Brazil; paulorbr@posgrad.lncc.br (P.R.R.); borges@lncc.br (F.B.)

**Keywords:** decentralized federated learning, secure aggregation, DC-nets, secret sharing, privacy

## Abstract

In the era of big data, millions and millions of data are generated every second by different types of devices. Training machine-learning models with these data has become increasingly common. However, the data used for training are often sensitive and may contain information such as medical, banking, or consumer records, for example. These data can cause problems in people’s lives if they are leaked and also incur sanctions for companies that leak personal information for any reason. In this context, Federated Learning emerges as a solution to the privacy of personal data. However, even when only the gradients of the local models are shared with the central server, some attacks can reconstruct user data, allowing a malicious server to violate the FL principle, which is to ensure the privacy of local data. We propose a secure aggregation protocol for Decentralized Federated Learning, which does not require a central server to orchestrate the aggregation process. To achieve this, we combined a Multi-Secret-Sharing scheme with a Dining Cryptographers Network. We validate the proposed protocol in simulations using the MNIST handwritten digits dataset. This protocol achieves results comparable to Federated Learning with the FedAvg protocol while adding a layer of privacy to the models. Furthermore, it obtains a timing performance that does not significantly affect the total training time, unlike protocols that use Homomorphic Encryption.

## 1. Introduction

Today’s common life is surrounded by a variety of Artificial Intelligence (AI) uses, such as financial systems, social networks, transportation, and search engines. This is pushed forward by the growing computational power of computers and even smartphones. A significant part of the population now has devices in their pocket capable of collecting and processing an enormous and diverse amount of data, which continues to increase as even more precise and sophisticated tools are demanded. The concern is that these data may be personal or sensitive, thus making the development of strategies to ensure the security and privacy of users’ data urgent.

In this context, Federated Learning poses an essential role as an emerging technique to mitigate the mentioned issues. Federated Learning (FL) [1] is a form of distributed machine learning that aims to ensure data privacy, where a set—called a federation—of devices train a global machine-learning model in a collaborative and distributed way.

This processing can be done in both centralized and decentralized versions of FL. In Centralized Federated Learning (CFL), a central server coordinates the training process, receiving model updates from participating devices, aggregating them, and distributing the global model to devices. On the other hand, in Decentralized Federated Learning (DFL), there is no central server. Instead, the devices communicate directly with each other in a peer-to-peer network, receiving model updates from their neighbors.

Federated Learning and the Internet of Things (IoT) have great synergy, as they can leverage the benefits of both concepts to develop intelligent, efficient, and secure applications. For example, FL can allow IoT devices to cooperate to learn models for anomaly detection, activity recognition, or image classification without sending their data to the cloud or a central server. This synergy can improve the performance, privacy, and scalability of IoT applications. Some IoT applications that can benefit from using DFL are:

- Anomaly detection in sensor networks: Sensors can cooperate to learn an anomaly detection model from their local data without revealing their locations or measured values. This can be useful for monitoring events like fires, floods, and earthquakes.
- Health: Wearable devices can collaborate to learn an activity recognition model from their sensor data without exposing personal or health information. This can be useful in providing users with personalized feedback, recommendations, or alerts.
- Image Classification in smart cameras: Smart cameras can cooperate to learn an image classification model from their visual data without sharing the captured images. This can be useful for applications like surveillance, facial recognition, and object detection.

One of the most positive aspects of FL is that participants do not have to share their raw data with other participants or the central server. Instead, each participant only shares their locally trained model. However, recent work has shown that from the gradients of the model, it is possible to conduct attacks that break the privacy of participants, such as the membership inference attack [2], attribute inference attack [3] and reconstruction of the data of the participants [4,5].

Secure aggregation in Federated Learning is a problem that aims to aggregate local models trained by different devices so that the attacks described in the previous paragraph are not carried out, i.e., no participant can access or infer information about the data or models of other participants.

Secret Sharing is a kind of cryptographic technique that can be used in multiparty computing, enabling a user to share a secret by dividing it into *n* parts and sharing it with *n* other users in a particular way that only by gathering the knowledge of a minimal number of users, could reconstruct the secret. Multi-secret sharing is a generalization of Secret Sharing, allowing users to share more than one secret at a time.

Dining Cryptographers Networks (DC-Nets) [6] is a communication technique that allows network participants to broadcast a message to the other participants while maintaining anonymity. DC-Nets also allows participants to aggregate their messages anonymously and securely.

Using DC-Nets in FL can be computationally expensive, as it allows the aggregation of only one message at a time, whereas in FL, the message is the whole machine-learning model. By using Secret Sharing and Multi-Secret Sharing directly in FL, it is not guaranteed that the participants will not access or infer information about the data or the models of the other participants since they will have access to the individual models.

However, by combining Multi-Secret Sharing and DC-Nets, a decentralized, secure aggregation protocol for FL can be built in a way that maintains the anonymity of the participants and ensures that they will have access only to the aggregated model and not to the individual models of the other participants.

This work aims to propose and implement a secure aggregation protocol based on DC-Nets and Multi-Secret Sharing in DFL. Furthermore, this work evaluates the performance, quality, and privacy of the protocol. It compares it with other methods of secure aggregation, such as Homomorphic Encryption and secret sum protocols, analyzing the advantages and disadvantages of each method in terms of communication, computation, security, and privacy.

The remainder of the work is organized as follows. Section 2 presents a non-extensive literature review, while Section 3 explains the background of the techniques used. Section 4 shows the proposed secure aggregation protocol and Section 5 the computational results. Section 6 discusses the results and main findings. Finally, Section 7 presents the conclusions and future work.

## 2. Related Work

There are several works that propose aggregation protocols for DFL. This section presents some of the existing works in the literature. Furthermore, we present some of the main works related to DC-Nets.

### 2.1. DFL without Privacy

In [7], a novel decentralized federated learning framework named BrainTorrent is introduced. BrainTorrent provides a peer-to-peer environment where different medical centers can collaborate and reap mutual advantages without the need to share their data. However, it should be noted that in this framework, the models are shared without encryption, making them vulnerable to the attacks discussed in the introduction.

The work [8] presents a general framework for DFL that performs local updates and inter-node communications periodically. The purpose of the work is to balance these elements, enhancing the efficiency of FL while working within the constraints of limited communication and computing resources. To improve communication efficiency, the authors employ the gossip compression scheme introduced in [9].

In [10], a segmented gossip approach protocol is proposed for DFL. In their protocol, participants divide the models into segments after training the local model and exchange them with other participants. At the same time, they also receive segments from other participants. The aggregation of local models is carried out only when all pull requests for the exchange of segments are fulfilled.

The authors in [11] propose a protocol that takes into account the layer-wise model discrepancy to adjust the aggregation interval. Relaxing the model aggregation frequency decreases the communication cost while maintaining good model performance.

### 2.2. DFL with Privacy

In their work [12], the authors utilize the Alternating Direction Method of Multiplier (ADMM) technique to achieve decentralized aggregation. This approach involves creating separate groups and restricting communication within each group. By controlling the communication between participants during each aggregation round, the authors aim to minimize the potential privacy leakage. To strike a balance between privacy and accuracy, the authors introduce a measure called a “gap”, which represents the number of iterations required for two devices to be in the same group. The authors demonstrate that the groups formed using these gap constraints are equivalent to a specific category of solvable balanced incomplete block design problems in combinatorial design theory [13].

In [14], a protocol is proposed based on proxy models and Differential Privacy. The proxy model has a common architecture for all participants and is updated from the local model using differential privacy. This allows efficient information exchange between participants while maintaining local models and data privacy. The referred work shows superior results when compared to existing methods.

Through Homomorphic Encryption, digital signatures can be used to provide a DFL scheme that guarantees the confidentiality, integrity, and correctness of models in the training process [15]. All training done is based on the proposed algorithm, Efficient and Verifiable Cipher-based Matrix Multiplication.

In [16], a decentralized coordinate descent algorithm is proposed that allows participants to learn local models completely decentralized and asynchronously. A Non-Private version with no privacy guarantee is proposed, and a private version that uses Differential Privacy to guarantee the privacy of participants’ data.

In [17], the authors introduce a hierarchical ring topology as a solution to address the centralization issues in the traditional training framework. They approach the construction of the ring as an optimization problem and propose an efficient heuristic algorithm to solve it. Additionally, they incorporate Differential Privacy techniques to ensure data privacy.

The works [18,19,20] use blockchain technology to guarantee the integrity and correctness of the data and models used in the training. In [18], participants conduct online training and release only partial models and metadata in unencrypted format. Refs [19,20] use Differential Privacy to protect gradients and models; however, in [19], a participant must solve a mathematical puzzle to perform the aggregation of the other participants’ local models, while in [20] the perturbed models go through a verification and signature scheme to prevent Poisoning Attacks, and at the end, the unperturbed models are split with Shamir’s Secret-Sharing method [21] and shared with some participants who finally aggregate and recover the global models.

Therefore, related works use Differential Privacy to guarantee the privacy of participants’ data, except for [20], which also uses Secret Sharing.

### 2.3. DC-Nets

David Chaum introduced a solution to the dining cryptographers problem in his work [6]. This solution, known as the Dining Cryptographers Network or DC-Net, enables a participant in a network to send a message to other participants while maintaining anonymity. In other words, if an attacker attempts to identify the sender of a message, it will be impossible to determine which participant sent it, as all participants have an equal probability of being the sender.

Later, several works sought to improve some aspects of DC-Net. In [22], the authors examine the effectiveness of the DC-Net protocol and introduce novel constructs that can detect and identify dishonest participants without requiring interaction. The article also addresses the limitations of the DC-Net protocol, including collision and interruption issues, and proposes potential solutions to these challenges. Refs [23,24] suggest the utilization of an Abelian finite group (F,+) in place of the XOR operation to present a multi-round approach to address the disruption problem. In their study, Ref [25] suggest a three-step method for integrating the DC-Net protocol into peer-to-peer networks, commonly used in blockchain applications to distribute transactions and blocks among participants. The initial phase involves a DC-Net with a group size of k, ensuring k-anonymity. Subsequent phases handle the transmission process within the peer-to-peer network. In addition, the researchers analyze the privacy and security aspects of the extension of the DC-Net protocol [26], which enables the fair delivery of messages of various lengths from multiple senders.

This work proposes the use of DC-Nets combined with a Multi-Secret-Sharing scheme. With these two primitives, we can obtain anonymity of the participants, efficiency in communication, and computational cost since the shared data will be, at most, the size of the model, while maintaining the privacy of the local models since each participant will only have access to the aggregated model.

## 3. Background

### 3.1. Federated Learning

#### 3.1.1. Centralized Federated Learning

Centralized Federated Learning (CFL) refers to an approach to machine learning where individual participants or devices maintain the privacy of their training data while collaboratively training a global model. In the context of FL implementation, two main entities exist: Participants and the Central Server. Furthermore, the FL process can be broken down into three distinct steps.

1. The server initializes the global model and sends it to each participant who updates his local model with the global model;
2. Each participant trains their model without the need to share their local data;
3. The server receives the models from each participant, adds them to the global model, and goes back to step 1.

**Figure 1:**
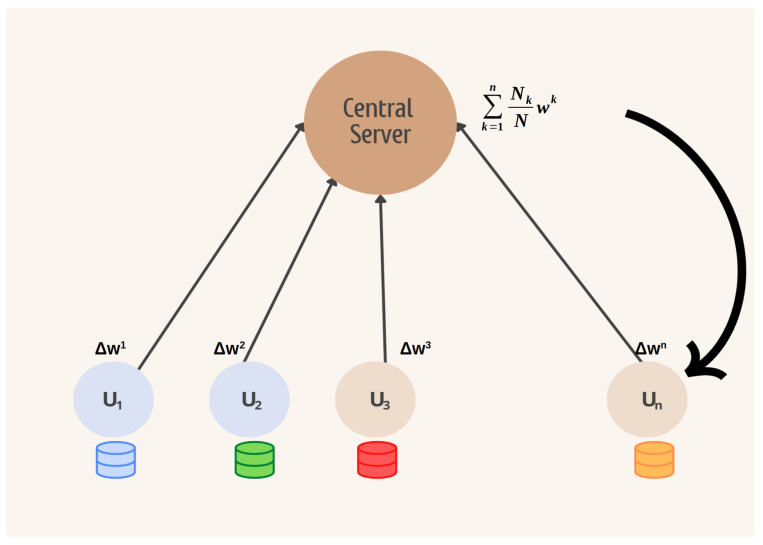
Centralized Federated Learning with FedAvg.

The third step mentioned above involves the execution of an aggregation algorithm on the server, a crucial component of FL. This aggregation algorithm is responsible for combining the individual models from the clients into a single model known as the global model. The most well-known algorithm for this purpose is FedAvg [1], which achieves aggregation by computing a weighted average of the local models. Subsequently, other algorithms have been developed based on FedAvg, aiming to improve the privacy of participants’ data and reduce communication costs. In this work, we also utilize FedAvg to ensure participants’ privacy through encryption. Figure 1 illustrates a basic aggregation round in FL using the FedAvg algorithm.

#### 3.1.2. Decentralized Federated Learning

Decentralized Federated Learning (DFL) is a variant of Federated Learning (FL) that removes the requirement for a central server to supervise the training process of an AI model. Instead, participating devices or organizations directly communicate with each other, creating a peer-to-peer network in which each node can independently initiate, join, or opt out of a training round. This approach improves the effectiveness, expandability, and resilience of FL, while also mitigating the potential risks associated with attacks or failures targeting the central server.

The DFL process can be outlined in the subsequent stages:

1. A training round is initiated by a node, which selects a subset of neighboring nodes at random to form a collaboration group.
2. Every node within the group individually trains its local model by utilizing its own private data.
3. The nodes within the group engage in the exchange of their local models with one another, employing a communication protocol that is both secure and efficient.
4. Every node combines the received models by employing a suitable aggregation algorithm and modifies its local model accordingly.
5. The initiator node terminates the training round and transmits a signal to the remaining nodes in the group.

**Figure 2:**
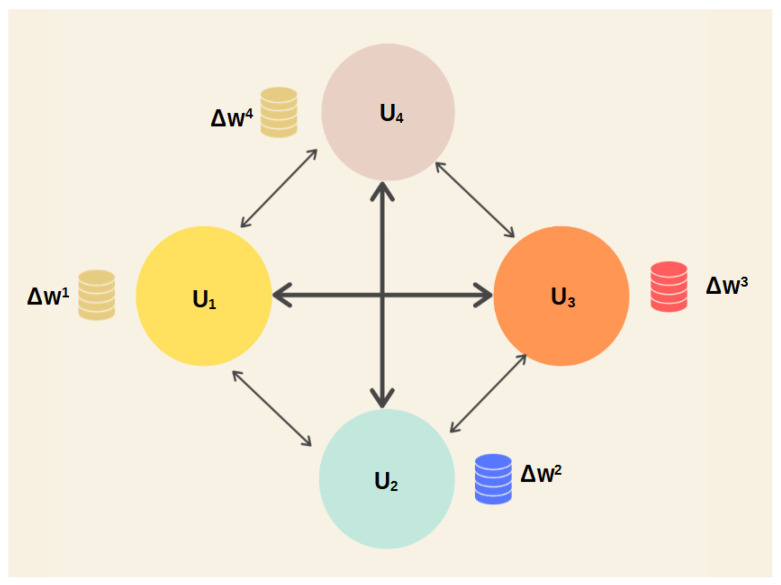
Decentralized Federated Learning.

Similar to CFL, DFL can be utilized in a range of contexts where FL is advantageous, including healthcare, finance, smart cities, the Internet of Things, and more. Nevertheless, DFL also introduces certain obstacles and constraints, such as the presence of diverse data and devices, the need for model synchronization and convergence, the selection and trustworthiness of nodes, and concerns regarding data and model privacy and security. To provide a visual representation of the process, Figure 2 depicts a simple aggregation round in FL employing the FedAvg algorithm.

When comparing DFL and CFL, several distinctions, advantages, and disadvantages can be identified.

- DFL is characterized by a higher level of decentralization compared to CFL, as it does not depend on a central server for training coordination. This feature enhances the autonomy and flexibility of participants while also mitigating the risks associated with potential bottlenecks or the failure of a single server.
- In terms of communication, DFL outperforms CFL by minimizing the quantity and size of messages transmitted between nodes. This can result in time, energy, and network resource savings, particularly in scenarios involving large amounts of data or multiple devices.
- In terms of fault tolerance, DFL outperforms CFL by allowing nodes to recover from errors or interruptions without compromising the training process. This capability enhances model quality and reliability, particularly in situations involving heterogeneous or non-IID data.
- Synchronization and convergence present greater challenges in DFL compared to CFL. In DFL, nodes must coordinate with each other to initiate and conclude training rounds. This aspect can complicate the management and assessment of the model’s advancement, particularly in situations involving devices that are dynamic or intermittent.
- In terms of selection and trust, DFL is more intricate than CFL because it necessitates nodes to form cooperative connections with other nodes. This process may encompass reputation, incentive, security, and privacy criteria, particularly in situations involving malicious or rogue organizations or devices.

### 3.2. Shamir’s Secret Sharing

**Figure 3:**
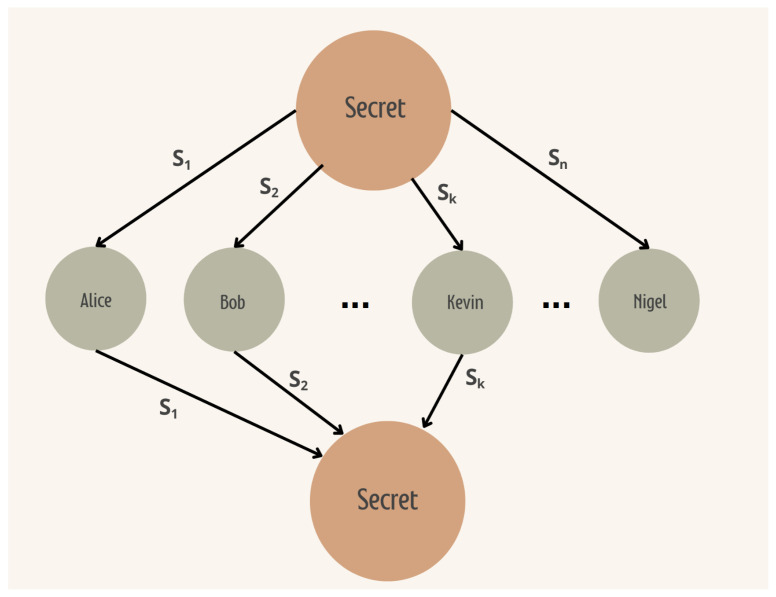
A (n,k) Secret-Sharing Protocol.

This Section introduces Shamir’s Secret-Sharing protocol [21]. Figure 3 shows the basic structure of a Secret-Sharing protocol. The secret is divided into *n* parts and shared among participants. With *k* parts of the secret, 1≤k≤n, the secret can be reconstructed. The protocol is as follows: One randomly samples numbers $a_{i}$ with i=1,...,k−1 and $a_{k-1}\neq 0$. Each $a_{i}$ will serve as a coefficient for the construction of the polynomial of degree k−1,

(1) $$f\left(x\right)=m+\sum_{i-1}^{k-1}a_{i}x^{i}.$$

It is easy to see that f(0)=m, where *m* is the secret to be shared. The distribution phase runs as follows: *n* distinct points are selected from f(x), i.e.,

(2) $$\left\{\left(x_{1},f\left(x_{1}\right)\right),\left(x_{2},f\left(x_{2}\right)\right),\cdots ,\left(x_{n},f\left(x_{n}\right)\right)\right\},$$

with $x_{i}\neq 0$. Anyone with a set of at least *k* points can reconstruct f(x) using Lagrange polynomial interpolation in the recovery phase and thus recover the secret f(0)=m. This protocol is usually called Shamir’s Secret Sharing (n,k).

### 3.3. Multi-Secret Sharing

Shamir’s Secret Sharing can be extended to a Multi-Secret Sharing protocol. Instead of randomly sampling the coefficients, they are set as the secrets, and a polynomial is constructed as

(3) $$f\left(x\right)=\sum_{i=0}^{k-1}s_{i}x^{i}.$$

The distribution phase is executed in a similar manner to Shamir’s Secret Sharing, while the recovery phase is different. During the secrets recovery phase, instead of obtaining only the constant term (i.e., P(0)) of the polynomial as in the standard Shamir Secret Sharing, we need to interpolate a polynomial to retrieve all its coefficients, which are the secrets. To accomplish this, we use Vandermonde interpolation, which involves solving a linear system where the coefficient matrix is a Vandermonde matrix. See Equation (4)

(4) $$\left[\begin{array}{cccc} 1 & x_{0} & x_{0}^{2} & \cdots x_{0}^{n}\\ 1 & x_{1} & x_{1}^{2} & \cdots x_{1}^{n}\\ 1 & x_{2} & x_{2}^{2} & \cdots x_{2}^{n}\\ 1 & \vdots & \vdots & \ddots \vdots \\ 1 & x_{n} & x_{n}^{2} & \cdots x_{n}^{n} \end{array}\right]\cdot \left[\begin{array}{c} a_{0}\\ a_{1}\\ a_{2}\\ \vdots \\ a_{n} \end{array}\right]=\left[\begin{array}{c} p(x_{0})\\ p(x_{1})\\ p(x_{2})\\ \vdots \\ p(x_{n}) \end{array}\right]$$

### 3.4. DC-Nets

This Section presents symmetric and asymmetric DC-Nets. In addition, it presents DC-Nets with Secret Sharing, which can be used in symmetric as well as asymmetric DC-Nets.

#### 3.4.1. Symmetric DC-Nets

In [6], David Chaum proposed a protocol to solve what he called the dining cryptographers problem. This protocol became known as the Dining Cryptographers Network, or DC-Net for short. DC-Net allows a network participant to broadcast a message to the other participants while keeping their anonymity, i.e., if a network attacker tries to identify the sender of a message, it will be concluded that all participants have an equal probability of being the sender. Suppose there is collusion between the attacker and some participants in the network. In that case, they will still conclude that all other participants (non-colluding participants) are equally likely to be the sender. Therefore, to identify a sender on a DC-Net, one would need N−1 participants to form a collusion, where *N* is the number of participants in the network.

**Figure 4:**
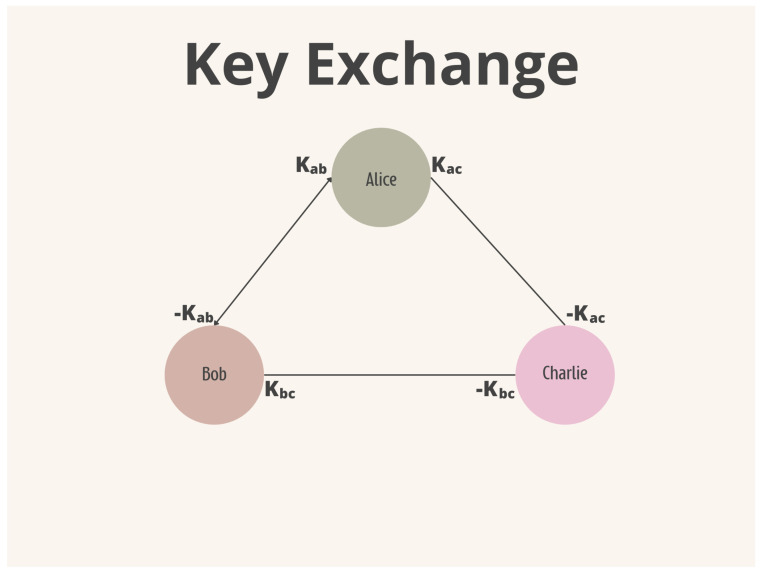
DC-Net Key Exchange.

**Figure 5:**
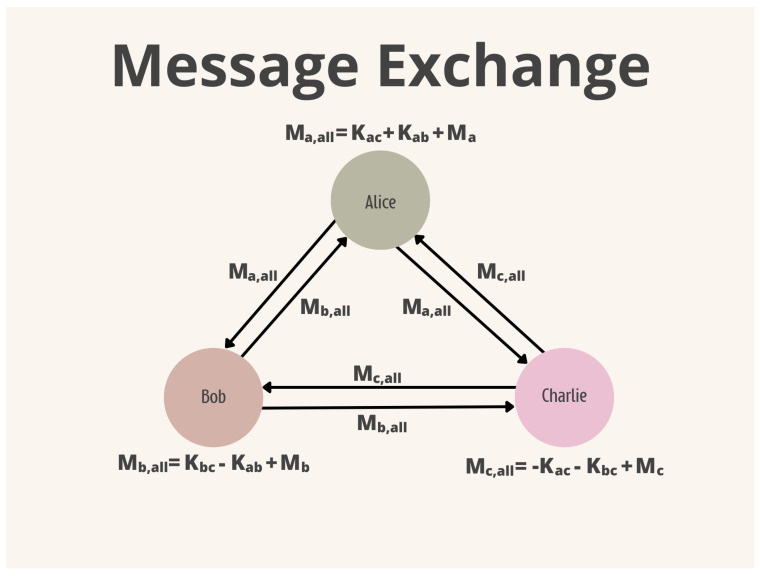
DC-Net Message Exchange.

There are some forms of topology in which a DC-Net can be constructed. Algorithm 1 presents a fully connected version, i.e., each participant needs to agree on a secret key with everyone else. This key is symmetric, changing only its sign. If participants *A* and *B* agree on the key *K*, they also need to agree on the sign of the key. For example, *A* will use *K*, hence *B* will use the switch −K. After the key agreement phase, each participant adds their keys to the message and sends the encrypted message to every participant. Finally, each participant adds their encrypted message with those of the other participants; the result will be the sum of all messages. Figure 4 and Figure 5 depict the key and message exchange phases.

As stated in [27], we have unconditional security if the keys are chosen truly at random; however, the keys can only be used once. In this work, we call it Symmetric DC-Net (SDC-Net) because in [27], the authors proposed an asymmetric version.
**Algorithm 1** SDC-net executed by user $u_{k}$.**Input:** Users $u_{i}$ for i=1,...,n with i≠k, message $m_{k}$.**Output:** Message $m_{out}$, which is the sum of all the messages of the participants. 1: Establish shared random secrets $s_{k,i}$ for each user $u_{i}$, with i≠k and $s_{k,i}+s_{i,k}=0$. 2: Calculate $M_{k}=m_{k}+\sum_{j=1,j\neq k}^{n}s_{k,j}$. 3: Send $M_{k}$ to $u_{i},\;\forall i\in \left\{1,...,n\right\}\setminus \left\{k\right\}$. 4: Receive $M_{i}$ from $u_{i},\;\forall i\in \left\{1,...,n\right\}\setminus \left\{k\right\}$. 5: Calculate $m_{out}=\sum_{j=1}^{n}M_{i}=\sum_{j=1}^{n}m_{i}$.

#### 3.4.2. Symmetric DC-Nets with Secret Sharing

**Figure 6:**
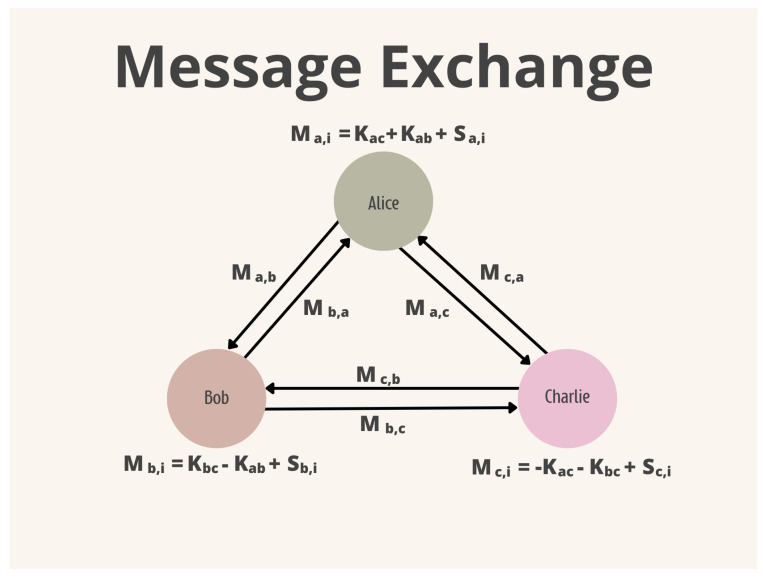
SDC-Net with Secret-Sharing Message Exchange.

Mödinger et al. [28] proposed a modified version of SDC-Net using Shamir’s Secret-Sharing protocol. Instead of a user sending the same encrypted message to all other users, he divides his message using (n,k) Shamir’s Secret Sharing (SSS) and sends a different share to each other user, as shown in Figure 6. In this way, to recover the message or the sum of all messages, it is enough for *k* of the users to send their part of the encrypted message to the server. Algorithm 2 presents the modified version of SDC-Net.
**Algorithm 2** Symmetric DC-Nets with Shamir’s Secret Sharing executed by user $u_{k}$.**Input:** Users $u_{i}$ for i=1,...,n with i≠k, message $m_{k}$.**Output:** Message $m_{k,out}$, the message share transmitted for this user. 1: Split $m_{k}$ into *n* parts $m_{k,i}$ for i=1,...,n with i≠k using a Secret-Sharing scheme. 2: Establish shared random secrets $s_{k,i}$ for each user $u_{i}$, with i≠k like the original DC-Net. 3: Calculate $M_{k,i}=m_{k,i}+\sum_{j=1,j\neq k}^{n}s_{k,j}$ for i=1,...,n. 4: Send $M_{k,i}$ for $u_{i},\;\forall i\in \left\{1,...,n\right\}\setminus \left\{k\right\}$. 5: Receive $M_{i,k}$ from $u_{i},\;\forall i\in \left\{1,...,n\right\}\setminus \left\{k\right\}$. 6: Calculate $m_{k,out}=\sum_{j=1}^{n}M_{i,k}$. 7: Send $m_{k,out}$ to all participants in the network. 8: Reconstruct $m_{out}$ after receiving k−1 other shares.

## 4. Proposed Protocol

**Figure 7:**
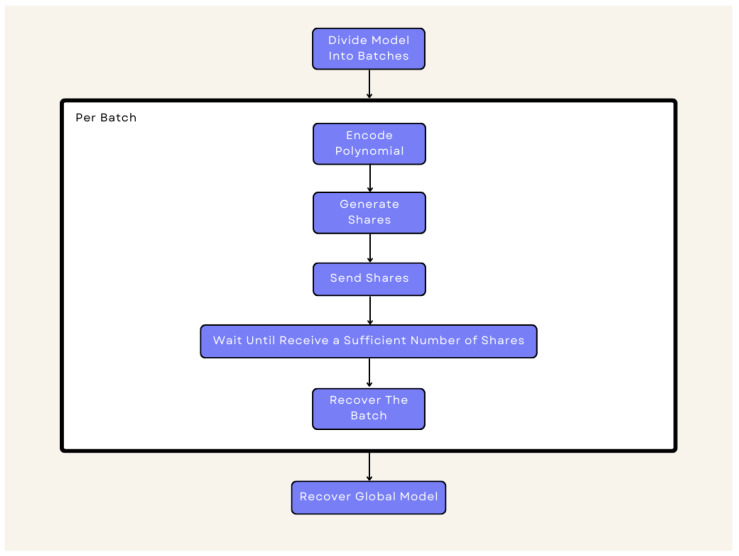
Proposed Protocol.

Figure 7 shows how it works. Each participant uses the Multi-Secret-Sharing scheme shown in Section 3.3 to generate the shares of the other participants. Suppose the number of participants is smaller than the batch size. In that case, participants will have to generate additional shares that they must share with other participants so that at the end of the communication round, they have sufficient shares to recover the polynomial. In the setup phase, users must agree on a prime *q* and an integer *t* that are the modulus for the coefficients and the degree of the polynomial, respectively. The users must also agree on a pairwise symmetric key changed only by its sign. For example, if user A uses *K* for user B, then B will use the key −K for user A. It is important to note that if the keys are chosen truly at random, we have unconditional security; however, the keys can only be used once. Therefore, for practical purposes, users can use a secure hash function *H* in conjunction with the key. Finally, each user $u_{i}$ must have a unique $ID_{i}$. It is assumed that the users are honest but curious, which is a standard threat model used in the FL literature [29].

### 4.1. Batch Division

**Figure 8:**
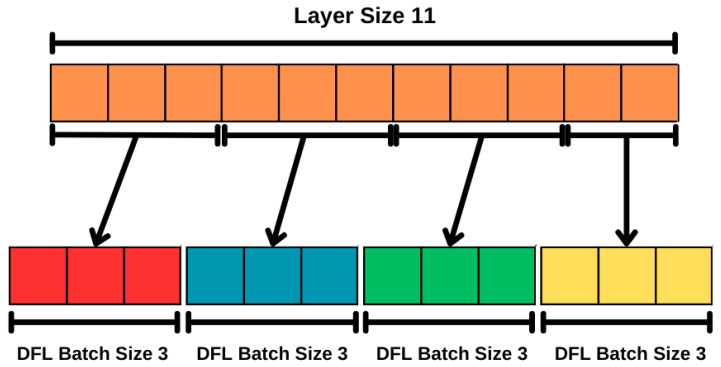
Batch Division with #layer_size=11 and #dfl_batch_size=3.

Using an idea similar to [30], in the proposed protocol, the model weights are divided into batches. This approach can reduce computational and communication overhead, thus leading to fewer costs. Figure 8 illustrates the batch division technique. With #dfl_batch_size defined, each model layer is divided into batches. If #layer_size is not a multiple of the #dfl_batch_size, the necessary amount of random numbers are sampled and added to the last batch. The number of random numbers sampled is given by:

(5) $$\left(\left\lceil \frac{\#layer\_size}{\#dfl\_batch\_size}\right\rceil \cdot \#dfl\_batch\_size\right)-\#layer\_size$$

### 4.2. Speeding Up Polynomial Interpolation

In the proposed protocol, we use the Multi-Secret-Sharing scheme shown in Section 3.3. Recall that, for the secrets to be recovered, it is necessary to solve a system of linear equations (of size #dfl_batch_size in our case) whose coefficient matrix is a Vandermond matrix. For example, solving a linear system using an LU Decomposition has a time complexity of $O(n^{3})$. However, as the #dfl_batch_size is fixed for each round of communication, as well as the abscissa of the points shared in the protocol, the decomposition is calculated only once, leaving only the resolution of upper and lower linear systems, which have a complexity of $O(n^{2})$. If #dfl_batch_size is fixed for all federated training and the abscissa of the points is also fixed, then each participant must compute the decomposition only once in the entire training. This drastically speeds up the secret recovery phase and, therefore, the whole protocol.

### 4.3. Protocol Phases

#### 4.3.1. Phase 1

1. After training the local model, users encode the local model weights as the polynomial coefficients in
  (6) $$f\left(x\right)=\sum_{i=0}^{k-1}w_{i}x^{i},$$
  with k=#dfl_batch_size. For further improvement, the SIMD paradigm can be used to encode more than one weight in a single coefficient.
2. Users calculate ($ID_{i},f\left(ID_{i}\right)$) for each user who participates in the network.
3. For all calculated pairs ($ID_{i},f\left(ID_{i}\right)$) encrypt as follows
  (7) $$M_{i}=f\left(ID_{i}\right)+\sum_{j=1,j\neq k}^{n}H(K_{i}||j)\;for\;i=1,...,n,$$
  where *j* is a counter for the aggregation round, a||b represents a concatenation of *a* and *b*, and *H* is a secure hash function.
  If the number of users is less than #dfl_batch_size, each user must generate extra points ($e_{I}D_{k},f\left(e_{I}D_{k}\right)$) for k=1,...,#users−#dfl_batch_size, and ensure that $\left\{e_{I}D_{k}\right\}\cap \left\{ID_{i}\right\}=⌀$.
4. Finally, send $M_{i}$ to the respective user $u_{i}$ and broadcast all ($e_{I}D_{k},f\left(e_{I}D_{k}\right)$), if it applies.

#### 4.3.2. Phase 2

1. After receiving $M_{i}$ from all users, calculate its share
  (8) $$m_{i}=\sum_{j=1,j\neq k}^{n}M_{i}\;for\;i=1,...,n.$$
2. Broadcast the share $m_{i}$ to all users.
3. After receiving at least *k* shares $m_{i}$ from users, reconstruct the polynomial (aggregated global model) using polynomial interpolation.

## 5. Results

This section presents the results obtained from the experiments to evaluate the proposed protocol. First, we ran experiments on the main modules of the protocol, i.e., batch division and encoding of model weights into polynomials, generation of shares, and recovery of the polynomial (polynomial interpolation). Subsequently, to compare our aggregation protocol with FedAvg, we performed experiments for two MLP scenarios, both using the MNIST handwritten digits dataset.

### 5.1. Execution Environment

The simulations were executed on a Dell computer with Ubuntu 22.04.3 LTS operating system, 12 GB of RAM, a 1.0 GHz Intel Core i5-1035G1 processor with eight cores, and 256 GB of solid-state drive (SSD).

### 5.2. Protocol Metrics

We evaluated the main parts of the proposed protocol, namely the generation of shares and recovery of the polynomial. Table 1 shows the computational complexity and Table 2 shows the average execution time of three main parts of the DFL protocol for different dlf_batch_sizes. As our protocol makes use of Honer’s method for polynomial evaluation in the Share Generation module and LU Decomposition for the Polynomial Recovery With LU module, the computational complexity of these modules is governed by the computational complexity of the methods used, i.e., O(n) for Honer method and $O(n^{3})$ for LU Decomposition. For the Polynomial Recovery with Precomputed LU module, we solve two triangular linear systems whose resolutions have complexity $O(n^{2})$. Share generation is the fastest part, taking less than 0.02 s for any dlf_batch_sizes. Polynomial recovery with LU is the slowest part, taking more than 2 s for a dlf_batch_sizes of 100. Polynomial Recovery with Precomputed LU is much faster than with LU.

### 5.3. Dataset

The MNIST dataset is a collection of handwritten digits widely used to train and test image processing systems. It contains 60,000 images in the training dataset and 10,000 images in the test dataset, each with 28×28 pixels in grayscale labeled with the corresponding digit from 0 to 9.

### 5.4. Scenarios Architectures

**Figure 9:**
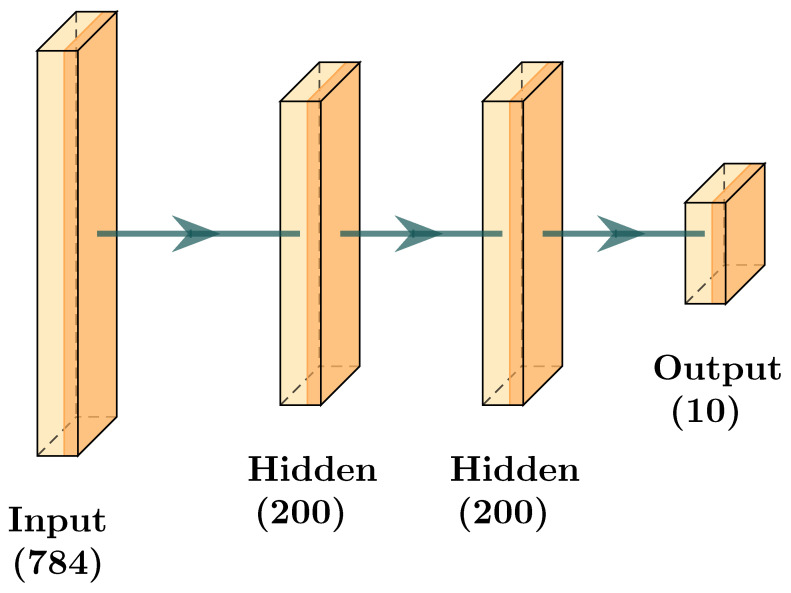
Image Representation of Scenario 1.

**Figure 10:**
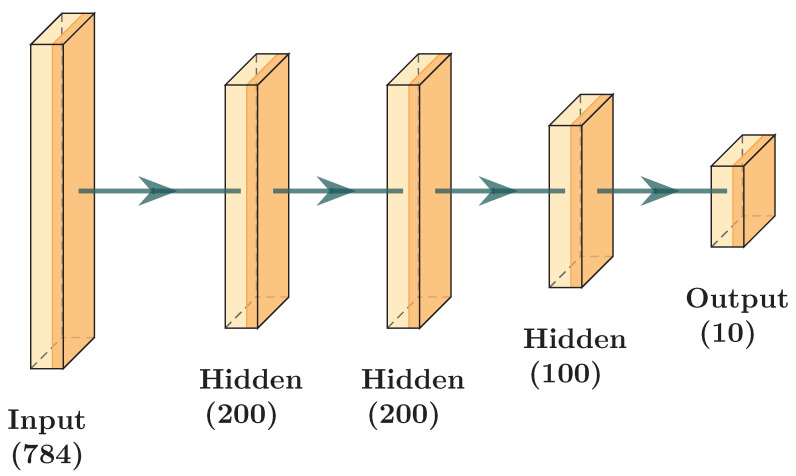
Image Representation of Scenario 2.

This section presents the architectures used in the experiments. The first scenario with architecture, as shown by Figure 9 and Table 3 was taken from [1]. The second scenario with architecture, as shown by Figure 10 and Table 4, is based on the first with the addition of a hidden layer. With these scenarios, we intend to compare the performance of a model trained using our aggregation protocol with a model trained using FedAvg.

The hyperparameters used in both scenarios are presented in Table 5:

For each scenario, two experiments were carried out. In the first experiment, Centralized Federated Learning was implemented using the FedAvg aggregation protocol [1]. In the second, Decentralized Federated Learning was carried out using the proposed aggregation protocol. It is known that distributed training, such as Federated Learning, can be affected by the heterogeneity, quality, and distribution of data among participants. However, the proposed protocol only adds a security layer to the aggregation phase. Theoretically, the outcome should be equivalent to an aggregation protocol that does not include the security layer. Furthermore, for the experiments, we used the MNIST Database of Handwritten Digits [31], which represents a simple, well-defined classification problem that is widely used as a benchmark.

#### Scenarios Results

Table 6 presents the metrics obtained for the two scenarios shown in this section for the standard Federated Learning aggregation with FedAvg [1] protocol and a Decentralized Federated Learning aggregation with the proposed protocol. Furthermore, the training dataset was split in a non-IID manner.

The results shown in Table 6 show that the proposed protocol does not significantly affect the performance of the model. This is a significant result because we only add a layer of security in the aggregation, and this layer, in theory, should have the same result as the aggregation without security. However, using the numerical method (LU Decomposition) in Polynomial Recovery phase may result in minor approximation errors, which may explain the slight variations in performance. In scenario 1, the proposed protocol achieved a lower loss than the same scenario with FedAvg. In scenario 2, the proposed protocol achieved a higher accuracy and precision than scenario 2 with FedAvg. The recall is the same for both scenarios. Scenario 1 performs better than scenario 2, with FedAvg and the proposed protocol. The difference of one hidden layer of 100 neurons does not affect the performance of the models significantly but may influence the complexity and the training time.

## 6. Discussion

Decentralized Federated Learning (DFL) is a machine-learning technique that allows multiple devices or users to train a model without sending the data to a central server. Despite its promise of data privacy, recent work shows that breaking the privacy of a given user’s data is possible using shared gradients. In this scenario, it is necessary to ensure that the aggregation of local models is conducted securely.

The main contribution of this work is a secure aggregation protocol for DFL. This protocol lets users share their local gradients securely and privately without revealing sensitive data. It has a minimal impact on the performance of the models compared to the FedAvg protocol. However, adding a security layer, which involves complex calculations, also increases computational cost. The proposed protocol is based on polynomial Secret Sharing and DC-Nets; thus, its main computational bottleneck is a polynomial interpolation. Fortunately, due to the protocol’s design, this step can be drastically sped up, making it computationally feasible. Although the #dfl_batch_size−1 is fixed, it depends directly on the number of model weights.

The proposed secure aggregation protocol for DFL based on Multi-Secret Sharing and DC-Nets is a relevant contribution to the field of FL, as it offers an efficient and reliable solution to the problem of gradient aggregation and can be applied in different scenarios and applications that involve distributed and sensitive data, such as health, finance, education, among others.

Two main lines of improvement of the proposed protocol can be highlighted. DC-Nets, and Polynomial Interpolation. DC-Nets provides sender anonymity and unconditional unobservability. However, when there are many participants, the system experiences slow data processing and increased computational time, resulting in higher delays. Therefore, using the k-anonymous group technique as presented in [25] can drastically reduce the communication cost. Furthermore, it is necessary to use techniques to prevent collision, disruption, and churn. Regarding polynomial interpolation, one can use fast solutions for Linear Systems with Vandermond Matrix [32,33,34] to improve the Polynomial Recovery phase.

Table 7 compares the proposed protocol with other protocols for DFL.

From a privacy perspective, users do not share their data directly with other users; only the encrypted shares (generated with the Multi-Secret-Sharing scheme) from each encrypted batch are sent using a DC-Net protocol. Therefore, an attacker would need the collusion of #dfl_batch_size−1 users to recover a batch from a user, which is inherent in the Multi-Secret-Sharing scheme. Furthermore, the collusion of #dfl_batch_size−1 is required to identify the share sender, which is inherent in the DC-Net protocol with multiple Secret Sharing.

## 7. Conclusions

This proposed work is a secure aggregation protocol for DFL based on Multi-Secret Sharing and DC-Nets. As far as we know, it is the first work that uses these two cryptographic primitives together to aid secure aggregation for DFL. We tested the efficiency of the model on the MNIST handwritten digits dataset. It was shown that the proposed protocol has minimal impact on the performance of the trained Deep Learning model while adding a layer of privacy to local models.

The proposed protocol ensures that clients do not share their data or gradients directly with other clients or the server. However, only the encrypted shares (generated with the Multi-Secret-Sharing scheme) of each encrypted batch are sent using DC-Nets. Therefore, an attacker would need collusion of #dfl_batch_size−1 clients to retrieve a batch from a client and to identify the sender of the share due to the Secret-Sharing scheme and the DC-Nets protocol. Thus, the protocol ensures theoretical security by information theory. Our protocol offers a high level of customer data privacy, similar to other protocols that use different secure aggregation techniques for DFL.

As future work, we intend to accelerate polynomial interpolation and evaluation using the techniques mentioned in the discussion section to use larger batches and increase the number of users in possible collusion. It is possible to speed up the entire protocol using SIMD techniques on the polynomial coefficients of the Secret-Sharing method. We can encode more than one weight in a single coefficient. This would reduce the number of batches required to transmit the entire model, thus reducing the communication cost. It is important to analyze the proposed protocol’s energy efficiency, especially to verify its performance and feasibility in IOT scenarios. It is also necessary to study ways to mitigate common attacks on FL, such as data and model Poisoning Attacks. Finally, we intend to simulate and analyze the behavior of our protocol in a more real network environment, i.e., one subject to delay and packet losses, for example. 

## Figures and Tables

**Table 1:** Computational Complexity for Main Protocol Modules Per Batch.

Protocol Parts	Computational Complexity
Share Generation	O(n)
Polynomial Recovery With LU	$O(n^{3})$
Polynomial Recovery Precomputed LU	$O(n^{2})$

**Table 2:** Time Metrics for Main Protocol Modules Per Batch.

Protocol Parts	DFL Batch Size 25 (s)	DFL Batch Size 50 (s)	DFL Batch Size 100 (s)
Share Generation	0.0007	0.0028	0.0130
Polynomial Recovery With LU	0.0409	0.2860	2.3229
Polynomial Recovery Precomputed LU	0.0028	0.0112	0.0517

Share Generation refers to the evaluation of a polynomial where the coefficients are the model weights in all IDs and extras points; Polynomial Recovery With LU and Precomputed LU refers to the resolution of linear systems with LU factorization and with precomputed LU factorization, respectively.

**Table 3:** Layers and Number of Neurons for Scenario 1.

Layer	Shape (Input, Output)
Linear	(784,200)
Linear	(200,200)
Linear	(200,200)
Linear	(200,10)

**Table 4:** Layers and Number of Neurons for Scenario 2.

Layer	Shape (Input, Output)
Linear	(784,200)
Linear	(200,200)
Linear	(200,200)
Linear	(200,100)
Linear	(100,10)

**Table 5:** Scenario Hyperparameters.

Hyperparameter	Value
Federation Participants	5
Aggregation Rounds	60
Number of Local Epochs	10
Batch Size	10
Learning Rate	0.01
Optimizer	SGD
Loss Criterion	Cross Entropy Loss
Initialization	Xavier

**Table 6:** Metrics for Each Scenario.

Scenario	Recall	Precision	Accuracy	Loss
Scenario 1 FedAvg	98.59%	98.60%	98.60%	0.0815
Scenario 1 Our Protocol	98.56%	98.57%	98.57%	0.0793
Scenario 2 FedAvg	98.44%	98.42%	98.47%	0.1057
Scenario 2 Our Protocol	98.44%	98.49%	98.50%	0.0866

**Table 7:** Protocol Comparison.

Protocol	Communication	Security Technique
Our protocol	Broadcast to All Neighbors	DC-Net and Secret-Sharing
[14]	Broadcast to All Neighbors	DP
[15]	Broadcast to All Neighbors	SHE
[16]	Broadcast to All Neighbors	DP
[18]	Send To One Neighbors	DP and Blockchain
[19]	Broadcast to All Neighbors	DP and Blockchain
[20]	Broadcast to an Aggregation Committee Nodes	DP and Secret-Sharing

## Data Availability

The dataset used was the MNIST Database of Handwritten Digits [31].

## Abbreviations

The following abbreviations are used in this manuscript:

AI	Artificial Intelligence
CFL	Centralized Federated Learning
DFL	Decentralized Federated Learning
DP	Differential Privacy
DC-Net	Dining Cryptographers Network
FedAvg	Federated Averaging
FL	Federated Learning
IID	Independent and Identically Distributed
LU	Lower and Upper Factorization
MLP	Multilayer Perceptron
MSS	Multi-Secret Sharing
SIMD	Single Instruction, Multiple Data
SDC-Net	Symmetric Dining Cryptographers Network
SHE	Symmetric Homomorphic Encryption
